# Effects of Picture Size Reduction and Blurring on Emotional Engagement

**DOI:** 10.1371/journal.pone.0013399

**Published:** 2010-10-14

**Authors:** Andrea De Cesarei, Maurizio Codispoti

**Affiliations:** Department of Psychology, University of Bologna, Bologna, Italy; University of Granada, Spain

## Abstract

The activity of basic motivational systems is reflected in emotional responses to arousing stimuli, such as natural pictures. The manipulation of picture properties such as size or detail allows for investigation into the extent to which separate emotional reactions are similarly modulated by perceptual changes, or, rather, may subserve different functions. Pursuing this line of research, the present study examined the effects of two types of perceptual degradation, namely picture size reduction and blurring, on emotional responses. Both manipulations reduced picture relevance and dampened affective modulation of skin conductance, possibly because of a reduced action preparation in response to degraded or remote pictures. However, the affective modulation of the startle reflex did not vary with picture degradation, suggesting that the identification of these degraded affective cues activated the neural circuits mediating appetitive or defensive motivation.

## Introduction

From an evolutionary point of view, detection of threats and life-sustaining opportunities allowed survival and promoted the development of specialized systems to spot and respond to significant stimuli. To this purpose, motivational systems modulate perceptual processes and emotional reactions to significant pleasant and unpleasant stimuli, building the basis of emotional behavior. In real environments, motivational systems allow efficient detection and an adaptive response (approach or withdrawal) to quickly changing environmental stimuli which vary in the degree of threat or positive opportunity they represent.

In a laboratory context, it has repeatedly been shown that emotional pictures engage the motivational systems and evoke a broad range of cortical, somatic, and autonomic reactions. For instance, skin conductance (SC) activity is more pronounced when viewing emotionally arousing (pleasant or unpleasant) pictures, compared to neutral pictures, covarying with the arousal dimension [1]–[3]. One interpretation is that skin conductance reflects action preparation, which is consistent with the role of the sympathetic system in arousing and engaging behavior in response to significant events.

On the other hand, the startle reflex has been used to indicate which motivational system, the appetitive or the defensive, is engaged [4]. When startle probes are administered in the context of picture perception, blink responses are reliably potentiated when viewing unpleasant pictures, and inhibited when viewing pleasant pictures, compared to neutral picture processing [5]–[6]. Interestingly, this modulation is more pronounced in the context of highly arousing stimuli and is absent for low-arousing stimuli which only activate these motivational systems weakly [1], [7].

The context of picture viewing allows for investigation into the extent to which specific factors may modulate the imminence of a visual scene and emotional reactions. For instance, it has been shown that previous experience with a scene, or brief picture exposure, modulates the relevance of a picture to the observer, with less pronounced emotional reactions for repeated or briefly-presented compared to novel or sustained pictures [8]–[11].

Pursuing the same line of research, recent studies investigated the effects of picture size on emotional perception [12]–[14]. Picture size is naturally linked to picture imminence, as larger sizes are associated to the perception of nearer stimuli, compared to smaller sizes [15]. It has been shown that larger picture sizes are associated with more pronounced differences in subjective ratings of valence and arousal between emotional and neutral pictures, as compared to smaller picture sizes. Moreover, this linear relationship is reflected in the emotional modulation of skin conductance [16].

In the picture viewing paradigm, the most arousing categories prompt the largest skin conductance responses as well as the most pronounced inhibition/potentiation of the startle reflex. Therefore, as the affective modulation of SC was observed to increase linearly with picture size, it could well be that the emotional modulation of the startle reflex varies with picture size. The first goal of the present study was to examine this possibility, by comparing the affective modulation of the startle reflex and skin conductance changes elicited in the context of viewing emotional scenes varying in picture size.

The finding that the affective modulation of skin conductance change varied with picture size supports the possibility that the size of an arousing picture modulates sympathetic reactions. However, as fine-grained details are lost when reducing picture size, it might also be that a fuzzy representation of a scene is not effective in activating action preparation, regardless of its retinal size or the type of degradation which was applied [13], [15]. Therefore, the present study aimed to extend previous results regarding the effects of picture size on the affective response to another type of manipulation, namely picture blurring, which shares some perceptual effects with size reduction (i.e., the loss of fine-grained details), but does not vary the visual angle subtended by the pictures.

In the present study, picture blurring was chosen to mimic the loss of details which happens with picture size reduction, as it reduces picture detail but does not change the visual angle subtended by the picture. If, as assumed by a “size-as-distance” hypothesis, which assumes that a smaller visual angle may be associated with the perception of a stimulus which is far away, retinal size modulates action preparation, then an effect on the emotional modulation of skin conductance should only be expected when the visual angle is changed. On the other hand (“size-as-detail” hypothesis), if visual detail determines action preparation, and eventually modulates skin conductance, then a similar dampening of the emotional modulation of skin conductance should be expected following either manipulation, with no role for the visual angle.

## Methods

### Ethics Statement

The study was approved by the Ethical Committee of the Department of Psychology at the University of Bologna, and a written informed consent was obtained by all participants.

### Participants

40 participants (20 females, mean age = 22.5, *SD* = 3.68) took part in the present study for a €10 compensation. Due to technical problems, some participants were excluded from the analysis of some measures. Final Ns were as follows: subjective ratings, *N* = 40; eyeblink EMG, *N* = 37; SC change, *N* = 38.

### Stimuli and equipment

A total of 100 pictures were selected from various sources, including the International Affective Picture System (IAPS) [17], representing contents which varied from highly arousing pleasant to neutral and highly arousing unpleasant. Pictures belonged to five categories (each *N* = 20): erotic couples, romantic couples, neutral people, threat, and mutilated bodies. Additionally, 10 additional neutral pictures were selected for a practice block. Pictures were adjusted to the same level of brightness and contrast [12].

### Manipulation parameters

In the intact condition, pictures were viewed from 1 meter and subtended a visual angle of 20.96° (horizontal)×15.66° (vertical). In addition to the intact original image, four versions of each of the 100 images were created, by either reducing size or blurring the original picture to one of two different levels. In the size reduction condition, pictures were scaled to either 33% or 12.5% of the original size, and pasted onto a gray background of the same brightness as the average of all the images. Visual angles subtended in each condition were 6.99×5.22° (33%), and 2.62×.96° (12.5%). In the blurring condition, a low-pass filter was applied to each color level of the original image [15]. The filtering function passed all spatial frequencies below a falloff value, and declined parabolically to a cutoff value (3·falloff value), above which all frequencies were filtered out. In the remainder, the filters will be described by their cutoff value. The low-pass cutoffs used were 168.6 and 63.9 cycles per image (cpi).

The startle probe consisted of a 50 ms white noise burst with an instantaneous rise and fall time, which was presented using stereo headphones at a 95 dB volume. Pictures were presented on a 19-inch monitor which was 1 m away from the participant, using E-Prime software [18]. A chinrest ensured that the distance remained constant within and across participants.

### Procedure

On arrival at the laboratory, participants signed an informed consent form which included the information that arousing scenes would be presented. Then the participant was accompanied to the experimental room and the electrodes were placed. When this was completed, the experimenter read the instructions aloud and the experiment began. At the end of the session, participants were debriefed regarding the aims of the experiment.

After a practice block, which comprised 10 neutral pictures, each participant viewed 100 pictures, equally distributed in terms of category and degradation condition. No picture was repeated. During each trial, a picture was presented for 6 seconds in the center of the screen. At a random interval of either 3350 or 4350 ms after the onset of the picture, the startle probe was presented [19]. After a blank screen lasting 3 seconds, the visual rating scales of valence, arousal and vividness were presented for 4 seconds each. After an interstimulus interval (ITI) lasting between 10 and 12 seconds, the next trial began. During ten distinct ITIs, a startle probe was delivered. The sequences were constructed so that the startle probe which was presented during the ITI did not systematically follow a specific stimulus content.

### Physiological response measurement

The startle EMG was recorded at 1000 Hz from two 4-mm Ag/Cl electrodes placed on the orbicularis muscle of the left eye [20]. The signal was amplified by 50000 and band-pass filtered between 13 and 1000 Hz using a Coulbourn V75-04 module, and integrated with a time constant of 100 ms using a Coulbourn V76-23 module. Eyeblink data were offline reduced by scoring, for each trial, the amplitude and latency of the startle eyeblink [21]-[22]. Trials where no response was detected were scored as zeros. Blink amplitudes were then converted to T-scores, based on the magnitude of the eyeblinks elicited during the ITI [23].

Skin conductance was recorded using two 7-mm Ag/Cl electrodes filled with 0.05-m NaCl Unibase paste, placed on the hypothenar eminence of the left palm. The signal was acquired using a Coulbourn V71-23 module which was calibrated before each session in order to detect activity in the range of 0–25 µS and sampled to 20 Hz. For 6 participants, who showed relatively high levels of skin conductance, amplifier gain was set in order to detect activity in the range of 0–49 µS. In data reduction, SC change was defined as the maximum change occurring from 1 to 4 seconds following picture onset, compared to the last second preceding picture onset. For statistical analysis, SC change data were normalized using a logarithmic function [24].

### Ratings

After each picture, participants were asked to rate valence and arousal of their emotional state, and the vividness of the picture. Vividness was defined as the amount of details in the picture, and was assessed using a modified SAM scale. In the vividness scale, the most neutral item of the SAM valence scale was varied in contrast, producing images which were progressively less visible [13]. All ratings were collected on 9-points ordinal scales.

### Data analysis

Physiological responses and SAM ratings were averaged across participants and experimental conditions. Data were submitted to an analysis of variance (ANOVA) with design 5 (picture degradation)×5 (picture category). Where appropriate, a Huynh-Feldt correction was applied to the degrees of freedom.

Additionally, an analysis of contrast was carried out to describe significant trends in the data [25]. As the two manipulations (picture category and degradation) comprised several levels, this approach was chosen as it allows for the identification of predictable trends in the data, which can be interpreted according to previous literature and research hypotheses. The order of levels for the picture category was: erotic couples, romantic couples, neutral people, threat, and mutilated bodies. In this way, a linear effect indicated patterns which varied in pleasantness, while quadratic effects described patterns which were more (or less) pronounced for arousing compared to neutral stimuli. For picture degradation, the order of levels was: highly filtered, mildly filtered, intact, intermediate size, and small size. In this way, a quadratic effect indicated patterns which were more pronounced for degraded compared to intact pictures. A separate analysis compared blurred to size-reduced pictures to examine the possibility that one of the two degradations had a more pronounced effect compared to the other.

## Results

### Physiological responses

#### Skin conductance change

**Figure 1:**
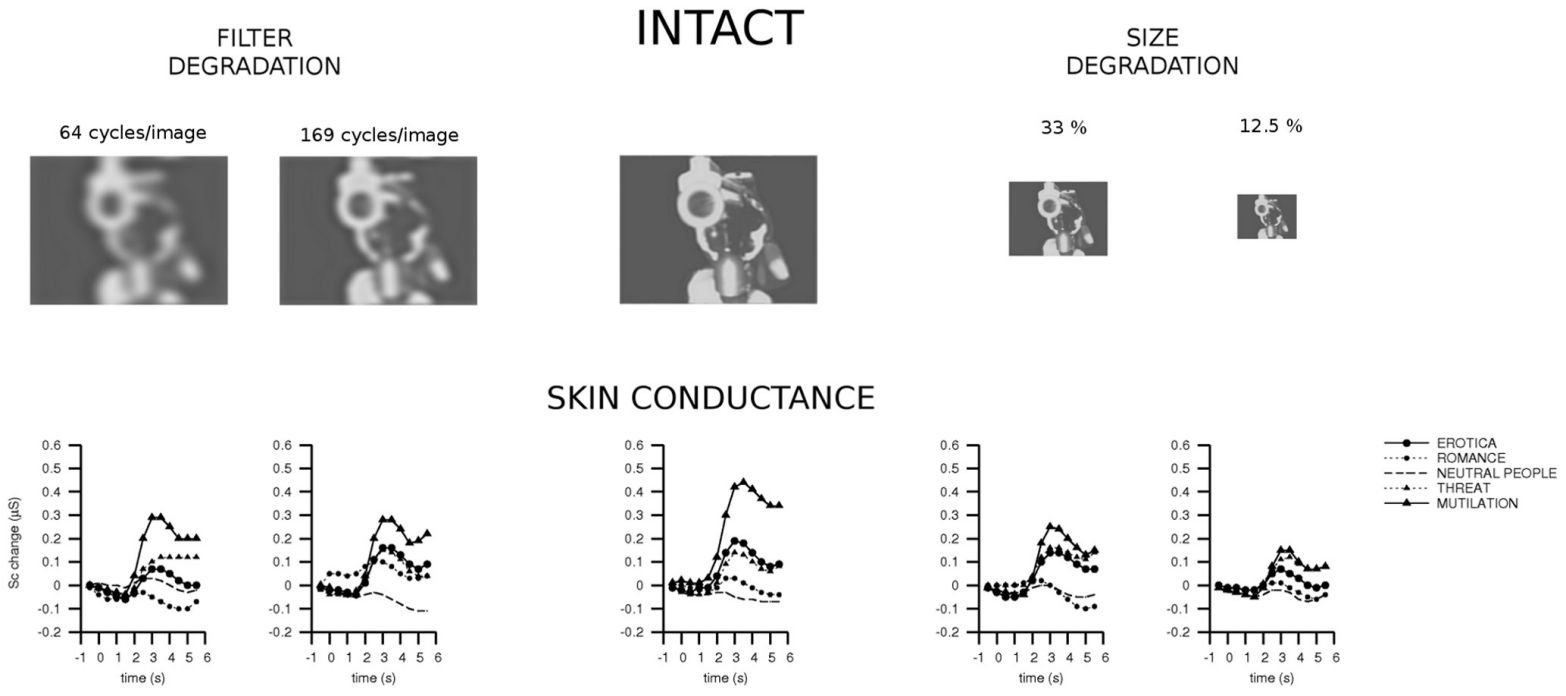
The effects of picture degradation on the affective modulation of skin conductance. In the central column, the original intact pictures are shown. At the sides, conditions where pictures were reduced by blurring (left) or by decreasing size (right) are presented. Affective modulation by highly arousing pleasant and unpleasant contents was observed in the intact condition, and this modulation was dampened by picture degradation. A more pronounced reduction of affective modulation was observed following picture size reduction compared to blurring. In the experiment, pictures were presented in color.

Picture category significantly modulated skin conductance change in the intact condition, *F*(4, 148) = 8.67, *p*<.001, *η^2^_p_* = .19, with more pronounced changes elicited by highly arousing compared to mildly arousing and neutral pictures, *p*s<.05 (see Figure 1). In the present study, pictures of mutilated bodies elicited a more pronounced SC change compared to erotic couples, *p*<.05, and scenes of threat elicited a more pronounced SC change compared to neutral pictures. This pattern of results was well described by a quadratic contrast (erotic couples > romantic couples > neutral people<threat < mutilated bodies), *F*(1, 37) = 14.38, *p*<.01, *η^2^_p_* = .28.

Affective modulation of skin conductance was reduced by stimulus degradation (see Figure 1), as revealed by the quadratic x quadratic interaction of picture category and degradation, *F*(1, 37) = 10.71, *p*<.01, *η^2^_p_* = .22. Replicating previous studies, size reduction dampened affective modulation of skin conductance, as indicated by the linear x quadratic interaction of size reduction (intact >33% size >12.5% size) and picture category, *F*(1, 37) = 10.77, *p*<.05, *η^2^_p_* = .23. Additionally, picture blurring reduced affective modulation of skin conductance, contrast of image blurring (intact > intermediate blurring > maximum blurring) x picture category, *F*(1, 37) = 6.53, *p*<.05, *η^2^_p_* = .15.

Affective modulation of skin conductance change was more dampened by size reduction compared to picture blurring (see Figure 1). This result was supported by a significant linear x quadratic interaction between type of degradation (blurring > size reduction) and picture category, *F*(1, 37) = 6.2, *p*<.05, *η^2^_p_* = .14. However, in all degradation conditions significant main effects of Category were observed, *F*s(4, 148)>3.13, *p*s<.05, *η^2^_p_*s>.08, and qualified by significant quadratic contrasts, *F*(1, 37)>6.24, *p*s<.05, *η^2^_p_*s>.14.

In short, skin conductance change was modulated by picture content in the intact condition, and this modulation was blunted by picture degradation. However, the effects of picture content were significant both in the size-reduced and in the filtered conditions. Finally, a more pronounced dampening of affective modulation of skin conductance was observed following size reduction compared to blurring.

#### Startle blink magnitude

**Figure 2:**
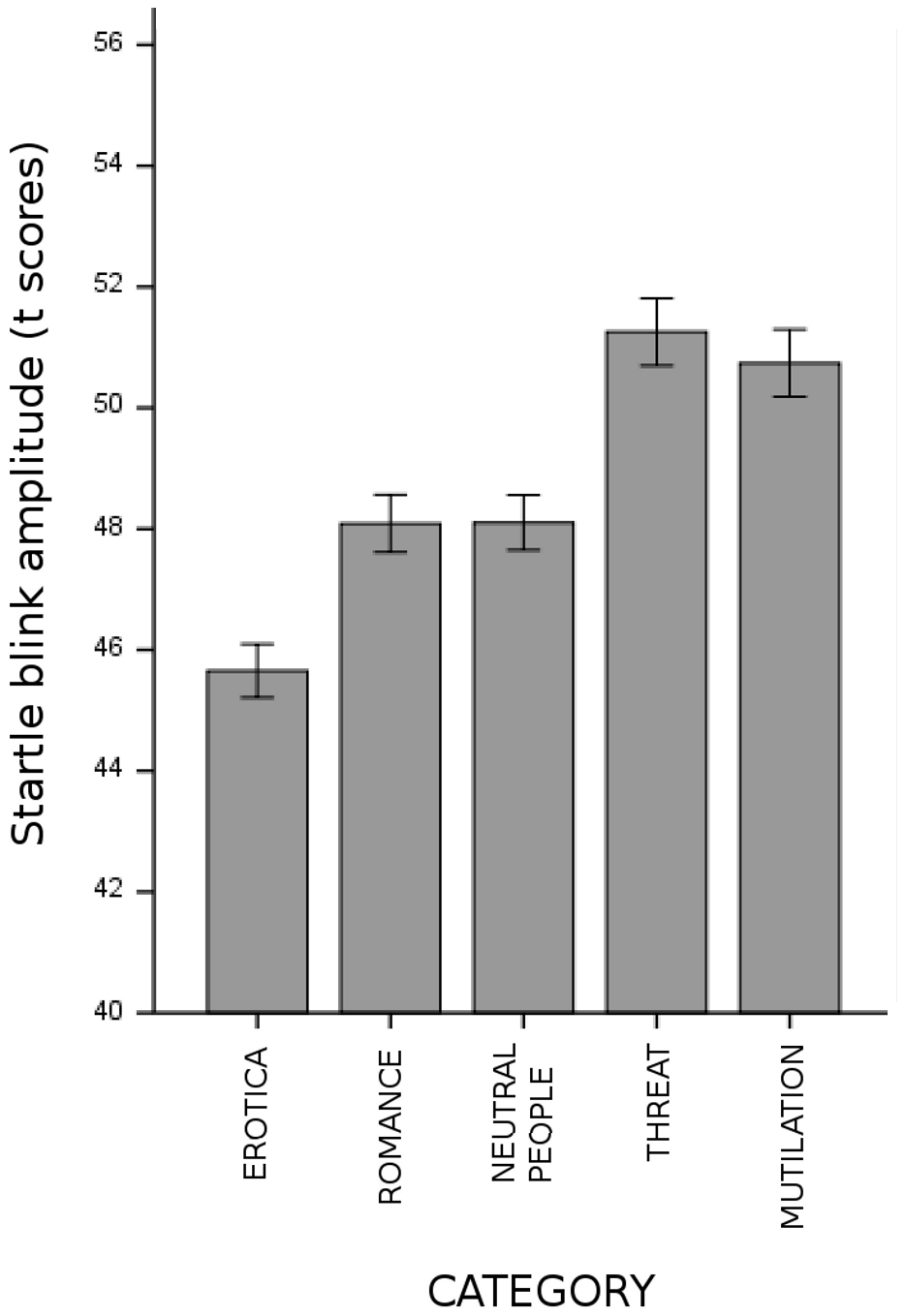
The effects of picture category on the startle eyeblink. Error bars represent standard errors. Magnitude of the startle eyeblink varied with picture valence, being potentiated by unpleasant contents, and inhibited by pictures of erotic couples.

Startle magnitude was modulated by picture category (see Figure 2), as indicated by the significant main effect of picture category *F*(4, 144) = 12.38, *p*<.001, *η^2^_p_* = .26 and by the linear contrast *F*(1, 36) = 26.98, *p*<.001, *η^2^_p_* = .43. Blinks elicited in the context of viewing pictures of erotic couples were significantly smaller than all other categories; blinks elicited while viewing pictures of threat and mutilation were significantly more pronounced compared to all other categories. No other comparisons reached significance.

No significant interaction of picture category and degradation was observed, which could indicate a modulation of this emotional effect caused by picture degradation. Across all categories, the magnitude of the startle eyeblink was higher when viewing degraded compared to intact pictures, picture degradation *F*(4, 144) = 3.14, *p*<.05, *η^2^_p_* = .08, quadratic contrast *F*(1, 36) = 7.73, *p*<.01, *η^2^_p_* = .18 (intact, M = 48.45; 169 cpi, M = 48.34; 64 cpi, M = 49.45; 33% size, M = 48.33; 12.5% size, M = 49.63). No significant differences between picture blurring and size reduction were observed.

A separate analysis which focused on raw startle data [20], replicated the main effect of picture valence, *F*(4, 144) = 21.18, *p*<.001, *η^2^_p_* = .37, qualified by the significant linear contrast *F*(1, 36) = 38.08, *p*<.001, *η^2^_p_* = .51. The startle magnitude was more pronounced in the context of degraded compared to intact pictures, *F*(4, 144) = 3.8, *p*<.01, *η^2^_p_* = .37, quadratic contrast *F*(1, 36) = 16.17, *p*<.001, *η^2^_p_* = .31. No interaction between picture degradation and picture category was observed in this analysis.

Taking these results together, startle blink magnitude was only modulated by picture content, with eyeblink potentiation for scenes of threat and mutilation as compared to neutral ones, and eyeblink inhibition for erotic compared to neutral pictures. Moreover, picture degradation resulted in an overall potentiation of the eyeblink.

### Subjective ratings

#### Valence

In the intact condition, valence ratings varied linearly with picture pleasantness (see Table 1), as indicated by the main effect of picture category *F*(4, 156) = 150.35, *p*<.001, *η^2^_p_* = .79, and by the linear contrast (erotic couples<romantic couples<neutral people<threat<mutilated bodies) *F*(1, 39) = 390.89, *p*<.001, *η^2^_p_* = .91.

**Table 1:** Affective ratings of valence.

	64 cycles/image	169 cycles/image	Intact	33% size	12.5%size	Total
Erotica	6.68 (1.43)	6.88 (1.31)	7.08 (1.33)	6.79 (1.34)	6.45 (1.53)	6.77 (1.39)
Romance	6.45 (1.49)	6.65 (1.52)	6.9 (1.56)	6.7 (1.6)	6.22 (1.47)	6.58 (1.53)
Neutral people	4.87 (1.31)	5.22 (1.52)	5.38 (1.51)	5.06 (1.31)	4.84 (1.12)	5.07 (1.35)
Threat	3.69 (1.65)	3.84 (1.7)	3.90 (1.80)	4.18 (1.85)	4.1 (1.6)	3.94 (1.72)
Mutilation	2.23 (1.38)	2.22 (1.5)	2.04 (1.15)	2.29 (1.42)	2.79 (1.46)	2.32 (1.38)
Total	4.79 (1.45)	4.96 (1.51)	5.06 (1.47)	5 (1.51)	4.88 (1.44)	4.94 (1.47)

The effects of picture content and degradation on ratings of valence. Values in brackets represent standard deviations.

Emotional modulation of valence ratings was reduced by picture degradation, picture degradation x picture category *F*(16, 624) = 7.22, *p*<.001, *η^2^_p_* = .16, linear x quadratic interaction between picture category and picture degradation, *F*(1, 39) = 48.59, *p*<.001, *η^2^_p_* = .56. Moreover, this reduction was more pronounced for size reduction compared to picture blurring, contrast of picture degradation (size reduction or blurring) and valence *F*(1, 39) = 19.98, *p*<.001, *η^2^_p_* = .34. Significant emotional effects were also observed in all degradation conditions, picture category *F*s(4, 156)>92.96, *p*s<.001, *η^2^_p_*s>.70, linear contrasts *F*s(1, 39) >173.36, *p*s<.001, *η^2^_p_*s>.82.

#### Arousal

A significant effect of stimulus category was observed on arousal ratings in the intact condition, *F*(4, 156) = 53.44, *p*<.001, *η^2^_p_* = .62, quadratic contrast (erotic couples > romantic couples > neutral people < threat < mutilated bodies) *F*(1, 39) = 188.06, *p*<.001, *η^2^_p_*  = .83. Highly arousing contents were associated with higher arousal ratings compared to all other categories, and mildly arousing scenes were rated as more arousing compared to neutral scenes (see Table 2). In the present study, pictures of mutilated bodies were rated as more arousing compared to erotic couples.

**Table 2:** Affective ratings of arousal.

	64 cycles/image	169 cycles/image	Intact	33% size	12.5% size	Total
Erotica	4.88 (2.15)	5.03 (2.25)	5.28 (2.21)	4.95 (2.2)	4.38 (2.27)	4.9 (2.22)
Romance	3.82 (2.3)	4.2 (2.29)	4.31 (2.26)	4.04 (2.2)	3.51 (2.04)	3.98 (2.22)
Neutral people	2.19 (1.52)	2.42 (1.74)	2.52 (1.79)	2.31 (1.64)	1.86 (1.26)	2.26 (1.59)
Threat	3.95 (2.33)	4.58 (2.44)	4.47 (2.38)	4.08 (2.25)	3.36 (2.17)	4.09 (2.31)
Mutilation	5.82 (2.53)	6.15 (2.3)	6.23 (2.42)	5.74 (2.44)	4.93 (2.49)	5.77 (2.44)
Total	4.13 (2.17)	4.48 (2.2)	4.56 (2.21)	4.22 (2.15)	3.61 (2.05)	4.2 (2.15)

The effects of picture content and degradation on ratings of arousal. Values in brackets represent standard deviations.

Picture degradation resulted in lower arousal ratings compared to the intact condition, picture degradation *F*(4, 156) = 54.56, *p*<.001, *η^2^_p_* = .58, quadratic contrast *F*(1, 39) = 93.6, *p*<.001, *η^2^_p_* = .71. Moreover, size reduction dampened arousal ratings more than picture blurring, as indicated by the comparison between blurred and size-reduced pictures, *F*(1, 39) = 48.29, *p*<.001, *η^2^_p_* = .55, and this reduction was similar for all picture categories. Significant effects of picture category were observed in all degradation conditions, *F*s(4, 156) >39.71, *p*s<.001, *η^2^_p_*s>.5, quadratic contrasts *F*s(1, 39) >101.76, *p*s<.001, *η^2^_p_*s>.72.

#### Vividness

**Figure 3:**
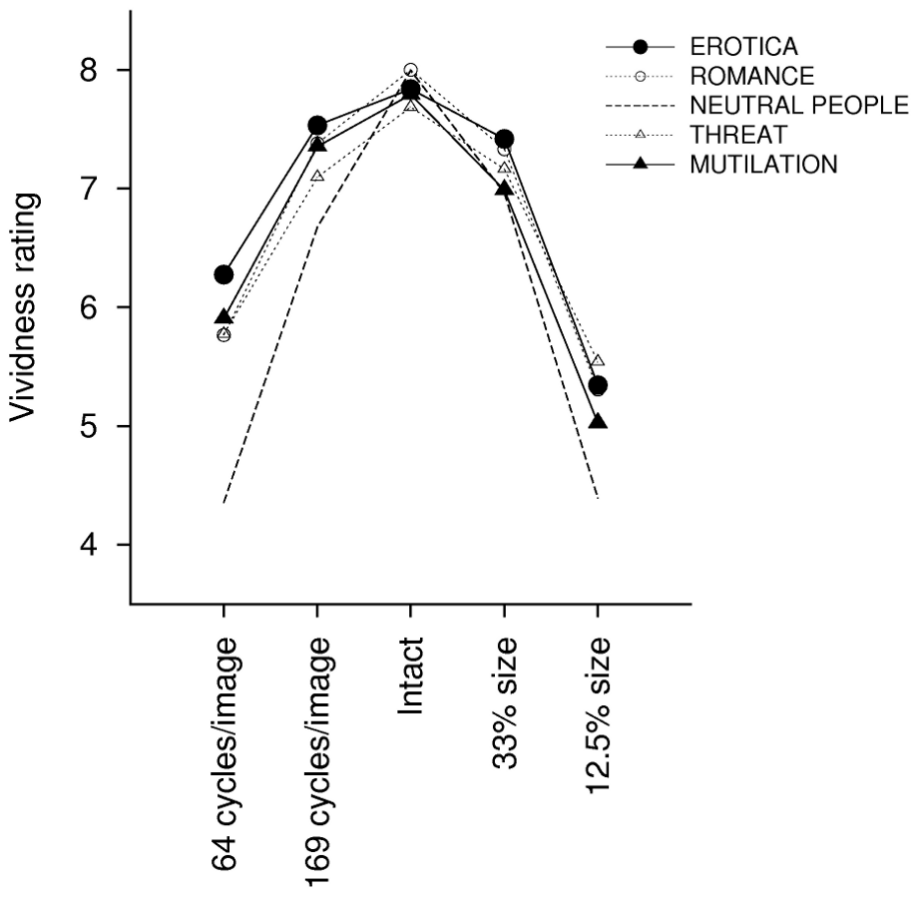
The effects of picture degradation on vividness ratings. Picture degradation reduced picture vividness, and this vividness loss was overall more pronounced following size reduction compared to picture blurring.

Degradation reduced picture vividness, *F*(4, 156) = 54.56, *p*<.001, *η^2^_p_* = .58. More specifically, a quadratic (highly filtered < mildly filtered < intact > intermediate size > small size) effect of picture degradation was observed, *F*(1, 39) = 246.83, *p*<.001, *η^2^_p_* = .86), indicating that ratings of vividness decreased with picture degradation (see Figure 3).

In the intact condition, no differences in vividness between pictures of different content were observed. However, picture degradation differently affected separate contents, picture content x degradation *F*(16, 624) = 12.48, *p*<.001, *η^2^_p_* = .24. In the smallest picture size, pictures of neutral people and mutilated bodies were rated as less vivid than all other categories, *p*s<.05. In the most blurred condition neutral pictures were rated as less vivid than all other categories, and pictures of erotic couples were rated as significantly more vivid than all other contents, *p*s<.05. As a result of this interaction, picture vividness was lower for the smallest compared to the most blurred pictures, *F*(1, 39) = 7.72, *p*<.01, *η^2^_p_* = .17.

## Discussion

In natural environments, the relevance and imminence of a life-threatening or life-sustaining stimulus modulate processing stages. In an evolutionary view, specialized motivational systems have developed so as to allow the adaptive perception and response of significant stimuli. In safe laboratory contexts, it has been reliably shown that viewing pictures of arousing natural scenes may engage motivational systems, resulting in specific behavioral and physiological changes [1].

Replicating previous results in the picture-viewing context, in the present study arousing picture contents modulated skin conductance change, and a reduction in picture size dampened the affective modulation of SC change. This result suggests that, even in a safe context such as the viewing of static pictures in the laboratory, the physiological mobilization persists as a preparatory state, and the size of emotional pictures modulates action preparation to relevant contents [16]. In terms of the functional significance of these autonomic changes and their role in action preparation and emotional behavior, it is interesting to note that Edelberg [26] suggested that skin conductance responses evoked by affective cues may be thermoregulatory responses, controlled primarily by the activation of hypothalamic and limbic centers. In particular, these responses may be adaptive for situations which, in the course of evolution, required motor activity and therefore resulted in increased body temperatures. This increase in the activity of sweat glands thus facilitates heat loss and maintenance of body temperature. One possible speculation is that, by decreasing body temperature, the activation of sweat glands may have facilitated survival in life-threatening contexts and the affective modulation of skin conductance during picture perception reflects this long-standing association between the emotional context and electrodermal activity.

A dampening of the emotional modulation of SC was observed following picture size reduction, image blurring, repeated picture presentation, and brief image exposure [8]–[11]. Altogether, these results suggest that the relevance of an affective cue, in the context of picture viewing, can be modulated by perceptual and contextual factors, and this can be reflected in action preparation in reaction to arousing scenes.

As the most arousing contents elicited the most pronounced emotional responses, and picture size reduction dampened affective modulation of SC, picture size reduction could have been expected to determine a less pronounced affective modulation of the startle reflex. However, this was not found, and affective modulation of the startle reflex was similar across all size conditions. Similarly, previous habituation studies have shown that the affective modulation of the startle reflex was preserved after several repetitions, whereas SC modulation habituated rapidly with stimulus repetition and was reinstated only when new pictures were presented [8], [27].

Taking these results together, it can be suggested that in the picture viewing context the motivational systems are engaged whenever a relevant content is identified, with little room for modulation by perceptual factors such as picture size or blurring. Previous studies showed that, whenever the content of a visual scene could be identified, central and peripheral measures of picture categorization and attention allocation reflected a preferential response to emotional contents [12], [13], [16]. The present study extends these results, suggesting that whenever the content of static complex scenes can be identified, motivational systems are engaged and this is reflected in the emotional modulation of the startle reflex in response to an auditory probe.

A slight potentiation of startle magnitude was observed for degraded as compared to intact pictures. It has been observed that the magnitude of the startle reflex elicited by an auditory probe is inhibited when the organism is attending to visual stimuli [28]–[29]. According, the present results might suggest that attentional allocation was reduced for degraded compared to intact pictures, possibly because degraded stimuli require less attentional resources to be processed. However, the results of a previous study [13] did not find any effect of picture size reduction on attentional capture by unattended emotional stimuli. Therefore, future studies might assess the degree to which attention allocation varies with picture size, using multiple indicators of attentional processing.

The present research examined affective reactions in a formerly unexplored condition, namely picture blurring, which decreased perceptual detail without affecting the visual angle subtended by the picture. In this respect, the present study demonstrated that picture blurring can dampen the affective modulation of a skin conductance change, suggesting that the loss of fine-grained details may reduce action preparation. However, the effects of picture blurring on the affective modulation of SC were less pronounced than the effects of picture size. Therefore, picture blurring did not entirely account for the dampening of the affective modulation of skin conductance which followed size reduction, and the present results suggest that both perceptual detail and visual angle contribute to the affective modulation of SC. Moreover, neither picture size nor picture blurring affected the emotional modulation of the startle reflex.

In the present study, stimuli which lacked of detail information were associated to a less pronounced affective modulation of SC. Interestingly, recent results [30] suggested that global image features, operationalized as low spatial frequencies (LSF), would play a more important role in the microgenesis of emotional response as compared to high spatial frequencies (HSF). As affective modulation of SC was dampened following elimination of high spatial frequencies, the present study suggest a role for HSF in affective modulation of SC. However, the present study did not aim at differentially assessing the contribution of LSF and HSF to emotional response. To this end, future studies might include a condition where the contribution of LSF is reduced, in order to investigate the role of high and low spatial frequencies in emotional response.

The present study provides clear data demonstrating that factors which reduce the affective modulation of one component of emotional response (i.e., skin conductance) do not necessarily affect the emotional modulation of other responses (e.g., the startle reflex). This result is fully consistent with the view that emotional response is made up of several components, which subserve different functions and are sensitive to different factors [31]. In particular, the present study showed that picture imminence, manipulated through its size, dampened affective modulation of SC but did not affect the emotional modulation of the startle reflex in response to an auditory probe.

In addition to SC and startle reflex, ratings of arousal and pleasantness were modulated by the affective value of pictures in this and previous studies [1]. In particular, emotional modulation of arousal ratings paralleled modulation of SC, whereas the patterns of affective modulation of pleasantness ratings and startle magnitude varied with picture valence. However, the manipulation of picture degradation had different effects on these subjective and physiological components of emotional response. Picture degradation dampened emotional modulation of subjective pleasantness, but not of startle magnitude. Moreover, emotional modulation of SC but not of arousal ratings was dampened for degraded compared to intact images. These dissociations suggest that, while separate emotional responses might show a similar pattern of modulation, they might vary in the sensitivity to a modulatory factor (e.g., picture degradation), reflecting the functions subserved by different components of the emotional response. Therefore it is important that when investigating the modulation of emotional response, specific indexes are chosen in light of their functional value, and multiple indexes are used to have a comprehensive picture of the modulation of emotional response.

The context of picture viewing allows for an investigation, in a safe laboratory context, of central and peripheral components of emotional reactions related to the engagement of motivational systems. However, the picture viewing context lacks the active call for action which is associated with encounters with real arousing stimuli. Recently, some studies examined dynamic situations in which picture size was continuously varied, producing the effect of stimuli moving towards or away from the observer [32]–[33]. In particular, Löw and colleagues [32] investigated a situation in which the size of drawings looming towards the observer signaled opportunities to respond and gain or lose money. The results of this study clearly showed that, in an active context, imminent stimuli inhibited overall magnitude and eliminated the affective modulation of the startle reflex, indicating maximum attentional capture elicited by the looming stimuli. Future studies could further explore how the emotional content of the stimulus and the call for action interact in order to modulate the engagement of motivational systems.

Taken together, the results of the present study suggest a dissociation between emotional reactions in the picture viewing context. On one hand, whenever a relevant picture content is identified, motivational systems might be engaged, resulting in an inhibition/potentiation of the startle reflex. On the other hand, perceptual degradation of emotional pictures, obtained either through picture size reduction or image blurring, dampened the affective modulation of subjective ratings and skin conductance, suggesting that stimulus content and perceptual quality may interact in modulating action preparation.

## References

[1] Bradley MM, Codispoti M, Cuthbert BN, Lang PJ (2001). Emotion and motivation I: Defensive and appetitive reactions in picture processing.. Emotion.

[2] Greenwald MK, Cook EW, Lang PJ (1989). Affective judgment and psychophysiological response: Dimensional covariation in the evaluation of pictorial stimuli.. J Psychophysiol.

[3] Winton WM, Putnam LE, Kraus RM (1984). Facial and autonomic manifestations of the dimensional structure of emotion.. J Exp Soc Psychol.

[4] Lang PJ, Bradley MM, Cuthbert BN (1990). Emotion, attention, and the startle reflex.. Psychological Review.

[5] Vrana SR, Spence EL, Lang PJ (1988). The startle probe response: a new measure of emotion?. J Abnorm Psychol.

[6] Bradley MM, Cuthbert BN, Lang PJ, Dawson ME, Schell AM, Boehmelt AH (1999). Affect and the startle reflex.. Startle modification: Implications for neuroscience, cognitive science, and clinical science.

[7] Cuthbert BN, Bradley MM, Lang PJ (1996). Probing picture perception: Activation and emotion.. Psychophysiology.

[8] Bradley MM, Lang PJ, Cuthbert BN (1993). Emotion, novelty, and the startle reflex: Habituation in humans.. Behav Neurosci.

[9] Codispoti M, Ferrari V, Bradley MM (2006). Repetitive picture processing: Autonomic and cortical correlates.. Brain Res.

[10] Codispoti M, Mazzetti M, Bradley MM (2009). Unmasking emotion: Exposure duration and emotional engagement.. Psychophysiology.

[11] Codispoti M, Ferrari V, De Cesarei A, Cardinale R (2006). Implicit and explicit categorization of natural scenes.. Prog Brain Res.

[12] De Cesarei A, Codispoti M (2006). When does size not matter? Effects of stimulus size on affective modulation.. Psychophysiology.

[13] De Cesarei A, Codispoti M (2008). Fuzzy picture processing: Effects of size reduction and blurring on emotional processing.. Emotion.

[14] Detenber BH, Reeves B (1996). A bio-informational theory of emotion: Motion and image size effects on viewers.. J Commun.

[15] Loftus GR, Harley EM (2005). Why is it easier to identify someone close than far away?. Psychon B Rev.

[16] Codispoti M, De Cesarei A (2007). Arousal and attention: Picture size and emotional reactions.. Psychophysiology.

[17] Lang PJ, Bradley MM, Cuthbert BN (2001). International affective picture system (IAPS): Instruction manual and affective ratings..

[18] Schneider W, Eschman A, Zuccolotto A (2002). E-prime user's guide..

[19] Bradley MM, Codispoti M, Lang PJ (2006). A multi-process account of startle modulation during affective perception.. Psychophysiology.

[20] Blumenthal TD, Cuthbert BN, Filion DL, Hackley S, Lipp O (2005). Guidelines for human startle eyeblink electromyographic studies.. Psychophysiology.

[21] Balaban MT, Losito BDG, Simons RF, Graham FK (1986). Off-line latency and amplitude scoring of the human reflex blink with Fortran IV.. Psychophysiology.

[22] Cook EW (2001). VPM reference manual..

[23] Bonnet M, Bradley MM, Lang PJ, Requin J (1995). Modulation of spinal reflexes: Arousal, pleasure, action.. Psychophysiology.

[24] Venables PH, Christie MJ, Martin I, Venables PH (1980). Electrodermal activity.. Techniques in Psychophysiology.

[25] Loftus GR (1996). Psychology will be a much better science when we change the way we analyze data.. Curr Dir Psychol Sci.

[26] Edelberg R, Greenfield NS, Sternbach RA (1972). Electrical activity of the skin.. Handbook of Psychophysiology.

[27] Ferrari V, Bradley MM, Codispoti M, Lang PJ (2011). Repetitive exposure: Brain and reflex measures of emotion and attention.. Psychophysiology.

[28] Anthony BJ, Graham FK (1985). Blink reflex modification by selective attention: Evidence for the modulation of ‘automatic’ processing.. Biol Psychol.

[29] Filion DL, Dawson ME, Schell AM (1998). The psychological significance of human startle eyeblink modification: a review.. Biol Psychol.

[30] Vuilleumier P, Armony JL, Driver J, Dolan RJ (2003). Distinct spatial frequency sensitivities for processing faces and emotional expressions.. Nat Neurosci.

[31] Lang PJ, Bradley MM, Cuthbert BN, Lang PJ, Simons RF, Balaban M (1997). Motivated attention: Affect, activation, and action.. Attention and orienting.

[32] Löw A, Lang PJ, Carson Smith J, Bradley MM (2008). Both predator and prey: Emotional arousal in threat and reward.. Emotion.

[33] Mühlberger A, Neumann R, Wieser MJ, Pauli P (2008). The impact of changes in spatial distance on emotional responses.. Emotion.

